# Supplementary material for: Switching of band inversion and topological surface states by charge density wave
Source: Nat Commun. 2020 May 18;11:2466. doi: 10.1038/s41467-020-16290-w (PMC7235022; doi:10.1038/s41467-020-16290-w)
Supplement: Supplementary file 1 — Supplementary Information [file 41467_2020_16290_MOESM1_ESM.pdf]

Switching of band inversion and topological surface states by charge density wave

Supplementary information

N. Mitsuishi *et al.*

Supplementary Note 1: **Crystal structure and Brillouin zone of VTe_2.**
Supplementary Note 2: **Physical properties of $V_{1-x}Ti_xTe_2$.**
Supplementary Note 3: **Band calculation of $V_{0.87}Ti_{0.13}Te_2$ in the normal 1*T* phase.**
Supplementary Note 4: **ARPES spectra and EDC/MDC plots along high-symmetry lines.**
Supplementary Note 5: **Domains in the monoclinic CDW 1*T*'' phase.**
Supplementary Note 6: **Temperature-dependent ARPES for $V_{0.90}Ti_{0.10}Te_2$**
Supplementary Note 7: **EDCs/MDCs along $\bar{K}-\bar{M}-\bar{K}$ for 1*T*-$V_{0.90}Ti_{0.10}Te_2$.**
Supplementary Note 8: **Setup of spin-resolved ARPES measurement**
Supplementary Note 9: **ARPES images and EDCs/MDCs along $\bar{K}_1-\bar{M}_1-\bar{K}_1/\bar{K}_1-\bar{M}_2-\bar{K}_2$ for 1*T*''-VTe_2.**
Supplementary Note 10: **Band calculation with V3*d* orbital weights.**
Supplementary Note 11: **CDW effects on Te5*p* orbitals.**
Supplementary Note 12: **Band unfolding in 1*T*''-VTe_2.**

Supplementary Note 1: Crystal structure and Brillouin zone of VTe_2.

Supplementary Figures 1a and b respectively show the crystal structure and Brillouin zone of VTe_2 in the normal 1*T* phase (trigonal, $P\bar{3}m1$). The conventional unit cell is indicated by the black lines (see also Fig. 1 in the main text). Supplementary Figures 1c and d display the case for the 1*T*" phase where the CDW long range order is fully considered (monoclinic, *C*2/*m*). This CDW exhibits (3×1×3) supercell structure. The superstructure along the stacking direction (*i.e.* the last term "3") stems from the successive shift of CDWs between the adjacent layers [1, 2].

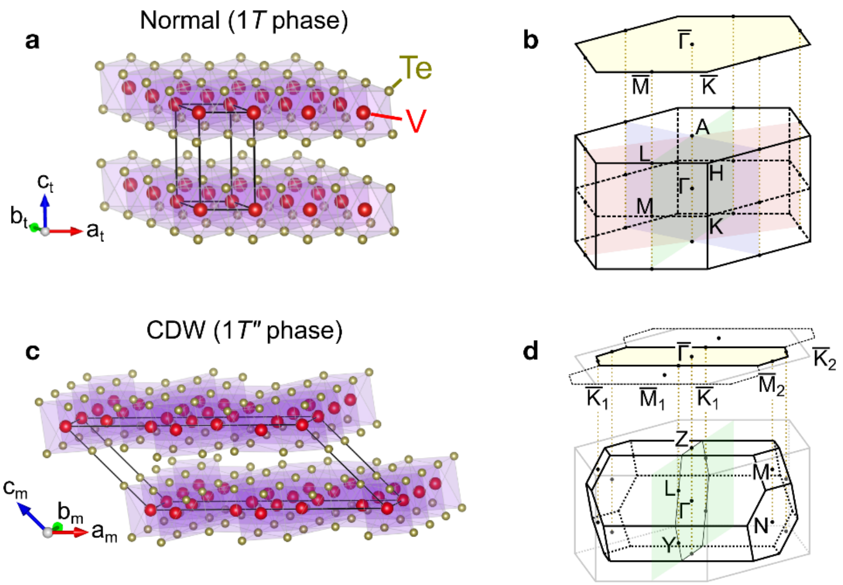

Supplementary Figure 1 | The details of crystal structure and Brillouin zone of VTe_2. a, Crystal structure in the high-temperature normal 1*T* phase. The conventional unit cell is indicated by the black lines. **b,** (0 0 1) surface 2D and bulk 3D BZs of 1*T*-VTe_2. Three color (RGB) planes represent the equivalent mirror planes. **c,** Crystal structure in the low-temperature CDW 1*T*" phase. **d,** (0 0 1) surface 2D and bulk 3D BZs of 1*T*"-VTe_2, superimposed on those in the 1*T* phase (light gray). The green plane indicates the mirror plane.

Supplementary Note 2: Physical properties of $V_{1-x}Ti_xTe_2$.

To grasp the overall phase diagram of $V_{1-x}Ti_xTe_2$, the x-dependent crystal structures and physical properties were thoroughly confirmed by using polycrystalline samples. The polycrystalline $V_{1-x}Ti_xTe_2$ were synthesized by solid state reaction. Stoichiometric mixture of V, Ti and Te powders ($1 - x_n : x_n$: 2) was pelletized and sealed in the evacuated quartz tube. The pellets were heated at 550 °C for 20 hours, and then cooled down to room temperature by furnace cooling. Supplementary Figure 2a shows the powder x-ray diffraction profiles ($30° \leq 2\theta \leq 33°$) of $V_{1-xn}Ti_{xn}Te_2$ at room temperature. Here we note that x_n, the nominal ratio of the raw material powders, does not precisely correspond to the actual x, due to some fluctuation of composition occurring during the growth. For lower x_n compositions, the two peaks assigned to $\bar{6}03$ and 310 reflections with $C2/m$ symmetry are clearly observed, representing the monoclinic 1T" phase. With increasing the Ti content, the peaks merge into a single peak corresponding to 101 reflection with $P\bar{3}m1$ structure, thus indicating that the simple trigonal 1T structure is obtained. Supplementary Figure 2b shows the temperature dependence of the electrical resistivity normalized by that at 300 K ($\rho(T)/\rho(300\ K)$). With increasing the Ti content, the anomaly corresponding to the phase transition becomes gradually suppressed to the lower temperature region. For $x_n \geq 0.3$, the anomaly is no longer observed in $\rho(T)/\rho(300\ K)$, indicating that the normal 1T phase becomes stabilized down to the lowest temperature. These results suggest that the 1T-1T" transition temperature in this system is favorably controllable by tuning the Ti doping level.

For the ARPES measurements, we synthesized high-quality single crystals of $V_{1-x}Ti_xTe_2$ for x = 0, 0.10, and 0.13, by the chemical vapor transport (CVT) method, as described in Methods. Here, the Ti contents (x) for respective samples were precisely determined by the EDX measurement, to perform the systematic x-dependent study. Supplementary Figure 2c shows the electrical resistivity for single crystalline $V_{0.90}Ti_{0.10}Te_2$ (x = 0.10). The anomaly corresponding to the CDW transition is discerned at around 300 K, which agrees with the temperature-dependent ARPES measurements shown in Supplementary Note 6. We note that the abrupt increase of resistivity on cooling below the transition temperature should be reflecting the disappearance of the Fermi surface at around $\bar{\mathrm{M}}_2$. On the other hand, there remain the Fermi surfaces at around $\bar{\mathrm{M}}_1$ and also around $\bar{\Gamma}$ points. The carriers on these highly warped Fermi surfaces should be responsible for keeping the conductivity until low temperature.

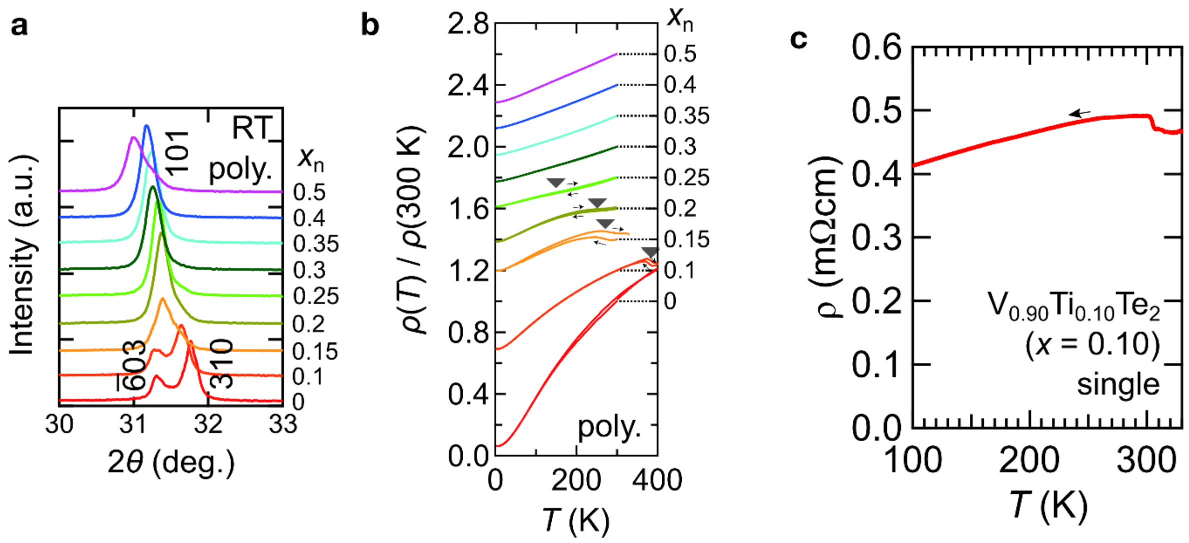

Supplementary Figure 2 | Physical properties of $V_{1-x}Ti_xTe_2$. a, Powder x-ray diffraction profiles recorded at room temperature. Here x_n is the nominal concentration of raw material powders and not precisely same as the actual x value of the grown samples. Miller indices based on the space group $C2/m$ ($\bar{6}03$ and 301) and $P\bar{3}m1$ (101) are depicted. **b,** Temperature dependence of normalized resistivity ($\rho(T)/\rho(300\text{ K})$). The anomaly corresponding to the phase transition are depicted by the gray triangle markers. **c,** Electrical resistivity for single crystalline $V_{0.90}Ti_{0.10}Te_2$ (x = 0.10). The anomaly corresponding to the CDW transition is discerned at around 300 K.

Supplementary Note 3: Band calculation of $V_{0.87}Ti_{0.13}Te_2$ in the normal 1*T* phase.

As discussed in the main text, we have calculated the electronic structure of 1*T*-$V_{0.87}Ti_{0.13}Te_2$ using virtual crystal approximation method. Supplementary Figure 3a displays the resulting 3D Fermi surface. Supplementary Figures 3b-f show the orbital-weighted band calculations in the global *xyz* setting (see also Figs. 4a and b in the main text).

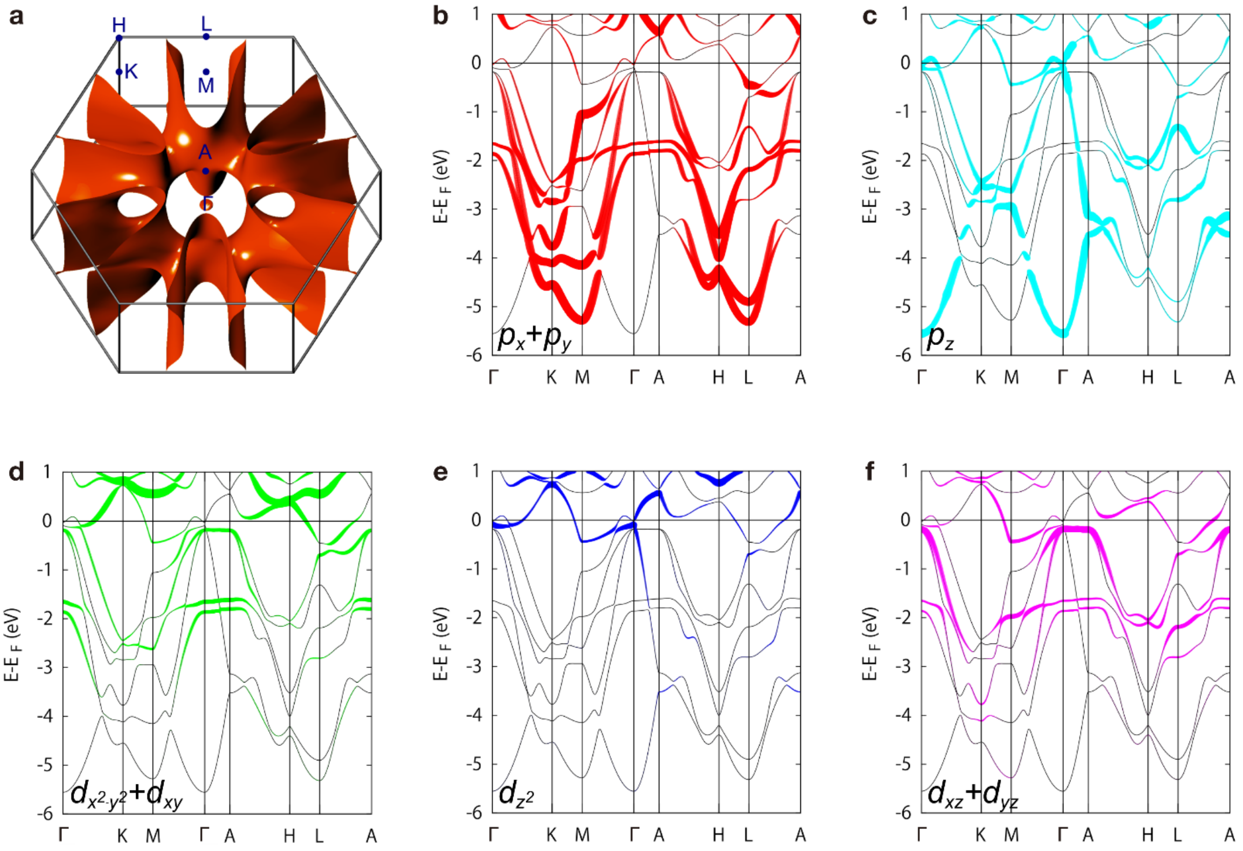

Supplementary Figure 3 | Calculated electronic structure of 1*T*-$V_{0.87}Ti_{0.13}Te_2$. a, 3D view of Fermi surface. **b-f,** Orbital-weighted band dispersions for p_x+p_y (**b**), p_z (**c**), $d_{x2+y2}+d_{xy}$ (**d**), d_{z2} (**e**), and $d_{xz}+d_{yz}$ (**f**).

Supplementary Note 4: ARPES spectra and EDC/MDC plots along high-symmetry lines.

Supplementary Figures 4a, d, and g respectively show the ARPES spectra of $1T$-$V_{0.90}Ti_{0.10}Te_2$ (350 K), $1T$''-$V_{0.90}Ti_{0.10}Te_2$ (20 K), and $1T$''-VTe_2 (15 K), taken with a He discharge lamp ($h\nu$ = 21.2 eV), which are identical to Figs. 3c, e, and g in the main text. The data of Supplementary Figs. 4a-c are divided by the Fermi-Dirac function convoluted with the Gaussian resolution function, to eliminate the thermal broadening of the Fermi cutoff. Note that the data in CDW phase includes the signal from all domains. The overlaid red (blue) markers indicate the EDC (MDC) peak positions. Supplementary Figures 4b, e, and h display the corresponding EDCs along $\bar{\Gamma}-\bar{K}$. For $V_{0.90}Ti_{0.10}Te_2$ in the normal state, we find no peak structure around the $\bar{K}$ point (see Supplementary Fig. 4c). On the other hand, for $1T$''-$V_{0.90}Ti_{0.10}Te_2$ and $1T$''-VTe_2, we detect continuous peak structures around E_B ~ 0.2 eV, corresponding to the anomalous flat band peculiar to the CDW $1T$'' phase (see also Supplementary Figs. 4f and i).

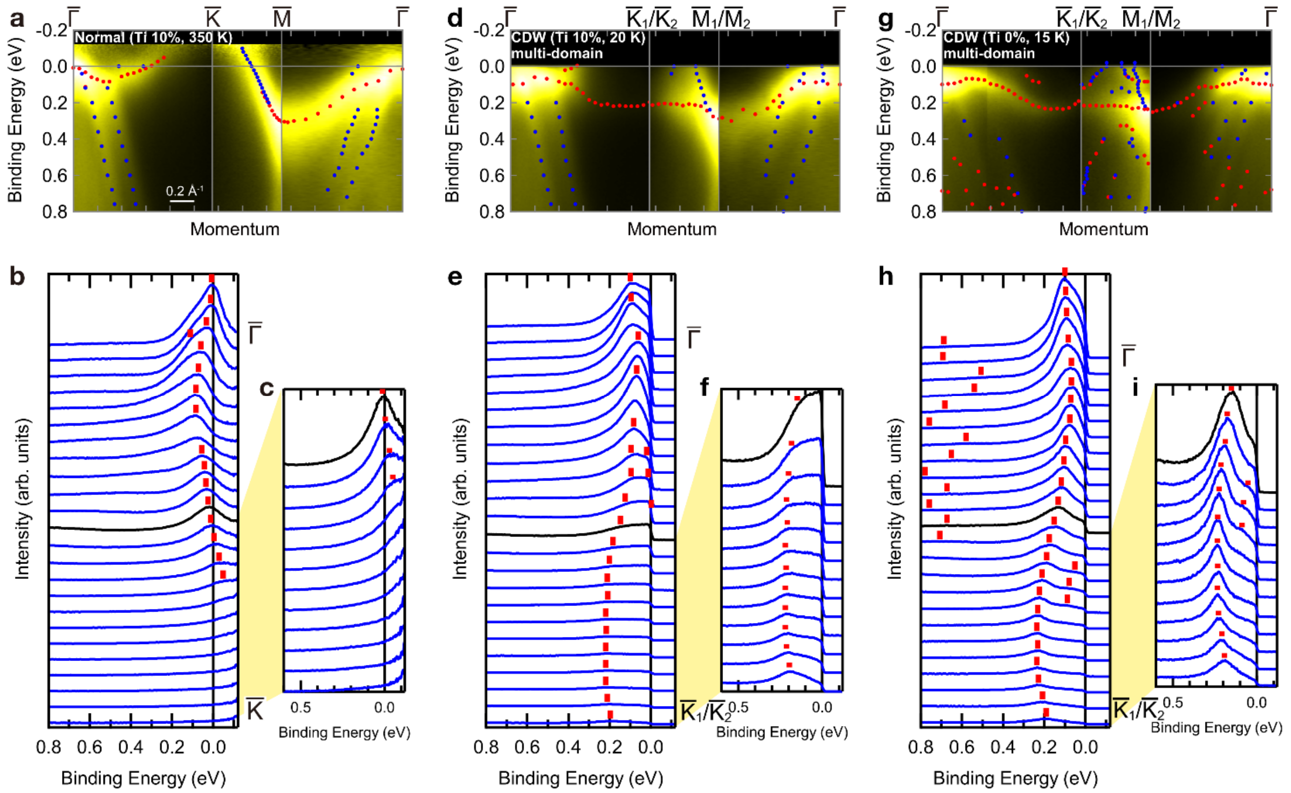

Supplementary Figure 4 | EDC/MDC plots along the high-symmetry lines with $h\nu$ = 21.2 eV (He discharge lamp). a, ARPES image of $1T$-$V_{0.90}Ti_{0.10}Te_2$ (350 K) with EDC (MDC) peak plots marked by red (blue) circles. **b,** EDCs of **a** along $\bar{\Gamma}-\bar{K}$ (integrated width: 0.05 Å^{-1}). **c,** Zoom-in of several EDCs around $\bar{K}$ point. The data of **a-c** are divided by the Fermi-Dirac function convoluted with the Gaussian resolution function **d-f,** Same as **a-c**, but for $1T$''-$V_{0.90}Ti_{0.10}Te_2$ (20 K, multi-domain). **g-i,** Same as **a-c**, but for $1T$''-VTe_2 (15 K, multi-domain).

Supplementary Note 5: Domains in the monoclinic CDW 1*T*'' phase.

In the monoclinic CDW 1*T*'' phase, there are six domains in total, due to the mixing of three 120 degree in-plane orientations and two monoclinic tilting orientations [1]. Supplementary Figure 5a shows one example of the polarization microscope image of 1*T*''-VTe_2 single crystal with (0 0 1) surface at room temperature. The straight stripes of bright and dark contrast with widths of 10-100 μm confirmed here reflect the domains with two different monoclinic orientations. On the other hand, in some cases, the orientation of the stripe patterns rotating by 120 degrees is observed in the length-scale of 10-1000 μm, as seen in Supplementary Fig. 5a. From these, we can identify the six domains in the 1*T*'' phase. Indeed, the stripe patterns vanish on heating the sample above 475 K, where the normal 1*T* phase is recovered.

The surface structure is also evaluated by a low-energy electron diffraction (LEED) measurement. Supplementary Figure 5b displays a LEED pattern of 1*T*''-VTe_2 cleaved and taken at room temperature (beam energy: 64 eV). We can assign the observed spots as depicted in Supplementary Fig. 5c. White circles represent the parent 1*T* spots, whereas red/green/blue circles are (3×1) CDW superstructures of three different in-plane domains. Here no extra spot is detected, thus the surface lattice ordering should be a (3×1) surface unit cell just as expected from its bulk (3×1×3) superstructure. We also note that similar surface structure is reported in the isostructural $NbTe_2$ [2] and $TaTe_2$ [3].

In the present ARPES study, the mixing of in-plane 120 degree domains makes it severely difficult to discuss the effect of the zigzag-chain CDW formation. For example, in Figs. 3e-h, the data includes the signal from all the domains since we use a He discharge lamp with the spot size of ~ 2 × 2 mm^2. To separately observe the 120-degree domains, we performed ARPES by employing synchrotron radiation with the smaller spot size of ~ 300 × 100 μm^2 (the data shown in Figs. 5a-e).

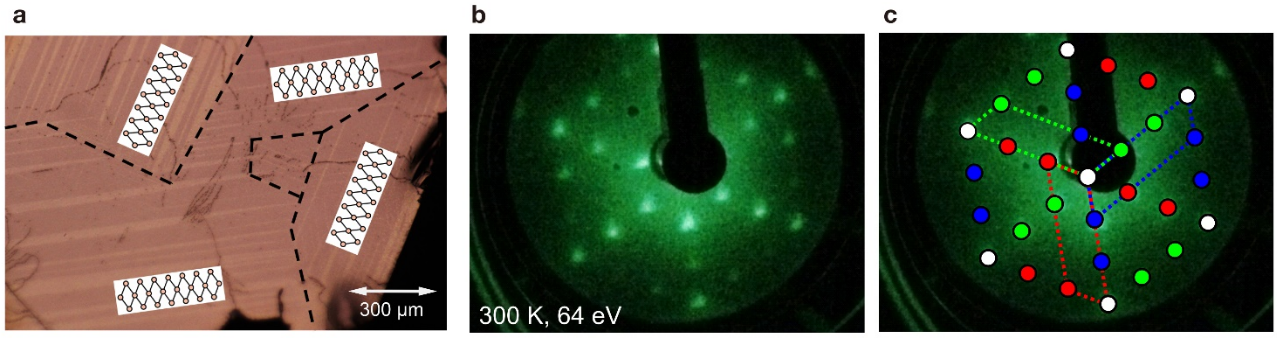

Supplementary Figure 5 | Domain structure of VTe_2 in the monoclinic CDW phase. a, Polarization microscope image at room temperature. 120 degree in-plane domains and their boundaries are respectively depicted by the cartoons and broken lines. **b,** LEED pattern taken with 64 eV beam energy at room temperature. **c,** Spot assignment of **b**. White circles represent the parent 1*T* spots, and red/green/blue circles show (3×1) CDW superstructure of different in-plane domains.

Supplementary Note 6: Temperature-dependent ARPES for $V_{0.90}Ti_{0.10}Te_2$.

We performed temperature-dependent ARPES measurement for Ti-doped $V_{0.90}Ti_{0.10}Te_2$ (transition temperature: ~ 280 K). Supplementary Figure 6a displays the ARPES image along $\bar{\mathrm{K}}-\bar{\mathrm{M}}-\bar{\mathrm{K}}$, recorded with a He discharge lamp ($h\nu$ = 21.2 eV) at several temperatures (cooling process). Note that the data are divided by the Fermi-Dirac function convoluted with the Gaussian resolution function, and in the CDW phase they include the signal from all the in-plane domains. Thus, here we simply use $1T$ notation instead of $1T$" (e.g. $\bar{\mathrm{M}}$ instead of $\bar{\mathrm{M}}_1/\bar{\mathrm{M}}_2$). On cooling, the photoelectron intensity reflecting the flat band emerges at around 250 K (E_B ~ 0.2 eV), indicating the evolution of CDW. This is also clearly confirmed from the temperature-dependent EDCs at the $\bar{\mathrm{K}}$ point (Supplementary Fig. 6b) and the Fermi momentum (k_F) of the V-shaped bulk band (Supplementary Fig. 6c). With further cooling, this flat band's peak structure sharpens and increases its intensity. We also note that the near-E_F intensity at k_F in Supplementary Fig. 6c is gradually suppressed on cooling below the CDW transition. Supplementary Figures 6d and e show the ARPES images respectively taken at 350 K and 20 K in a wider energy region (Supplementary Fig. 6d is identical to Fig. 4e in the main text). We clearly observe the lower branch of the Dirac surface state at both temperatures.

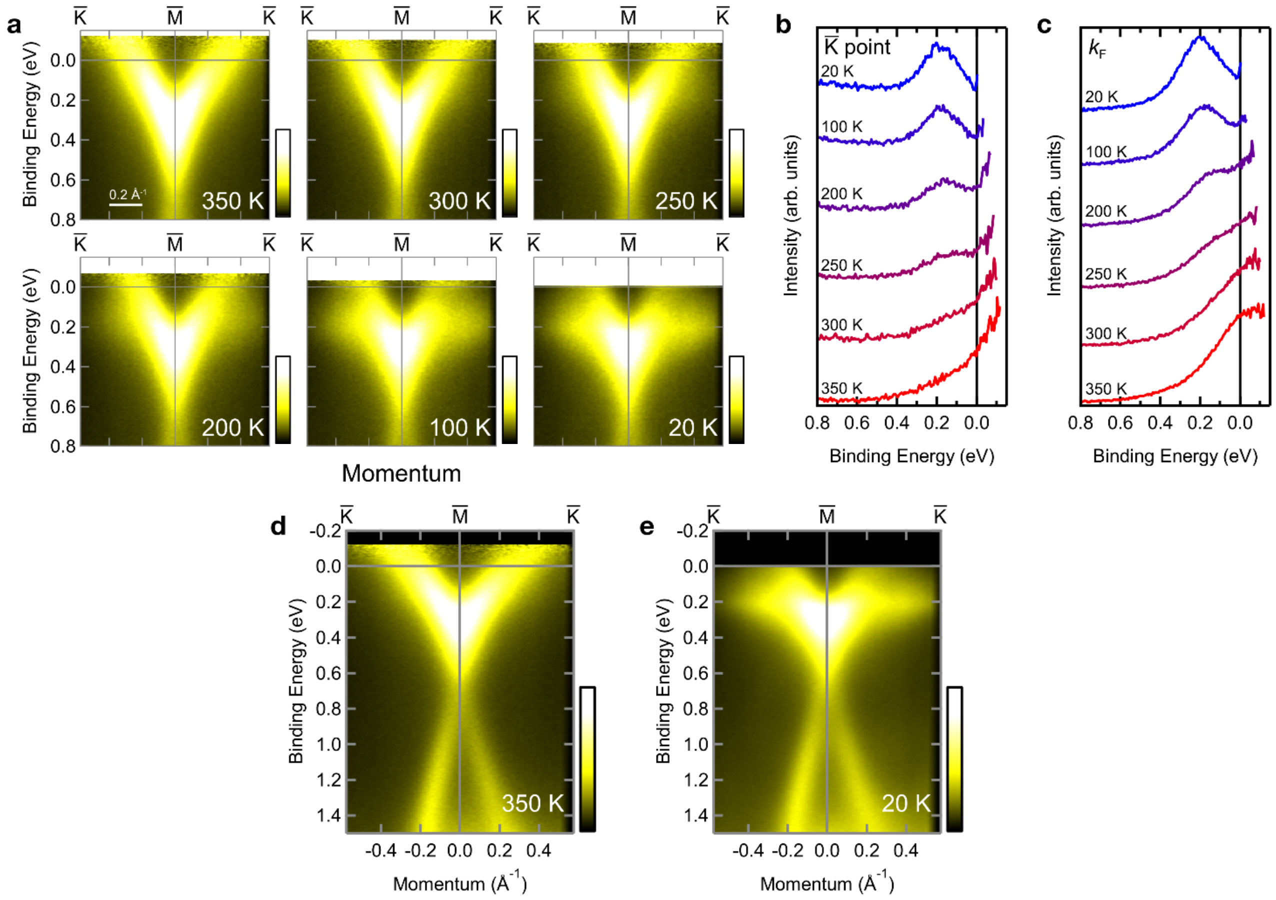

Supplementary Figure 6 | Temperature-dependent ARPES for $V_{0.90}Ti_{0.10}Te_2$. a, ARPES images along $\overline{\mathrm{K}} - \overline{\mathrm{M}} - \overline{\mathrm{K}}$ taken with a He discharge lamp ($h\nu$ = 21.2 eV) at several temperatures (350, 300, 250, 200, 100, 20 K). **b,** EDCs of **a** at $\overline{\mathrm{K}}$ point. **c,** EDCs of **a** at the Fermi momentum of the V-shaped band. **d, e,** ARPES images in a larger energy window taken at 350 K (**d**) and 20 K (**e**).

Supplementary Note 7: EDCs/MDCs along $\bar{\mathrm{K}}-\bar{\mathrm{M}}-\bar{\mathrm{K}}$ for 1*T*-$V_{0.90}Ti_{0.10}Te_2$.

Supplementary Figure 7 displays EDCs/MDCs with peak plots along $\bar{\mathrm{K}}-\bar{\mathrm{M}}-\bar{\mathrm{K}}$ for 1*T*-$V_{0.90}Ti_{0.10}Te_2$, corresponding to Fig. 4 in the main text. Supplementary Figures 7a and b respectively show EDCs and MDCs taken with a He discharge lamp ($h\nu$ = 21.2 eV, 350 K, see Fig. 4e). The energy positions of the bottom of bulk band A (E_B ~ 0.30 eV) and the top of bulk band B (~ 0.90 eV) are evaluated from EDCs, and that of the Dirac point (~ 0.66 eV) from MDCs. Supplementary Figures 7c-h show EDCs and MDCs recorded at 320 K with $h\nu$ = 63 (c, d), 61.5 (e, f), and 69 eV (g, h) circular polarization light, respectively corresponding to Figs. 4h, 4i, and 4j in the main text.

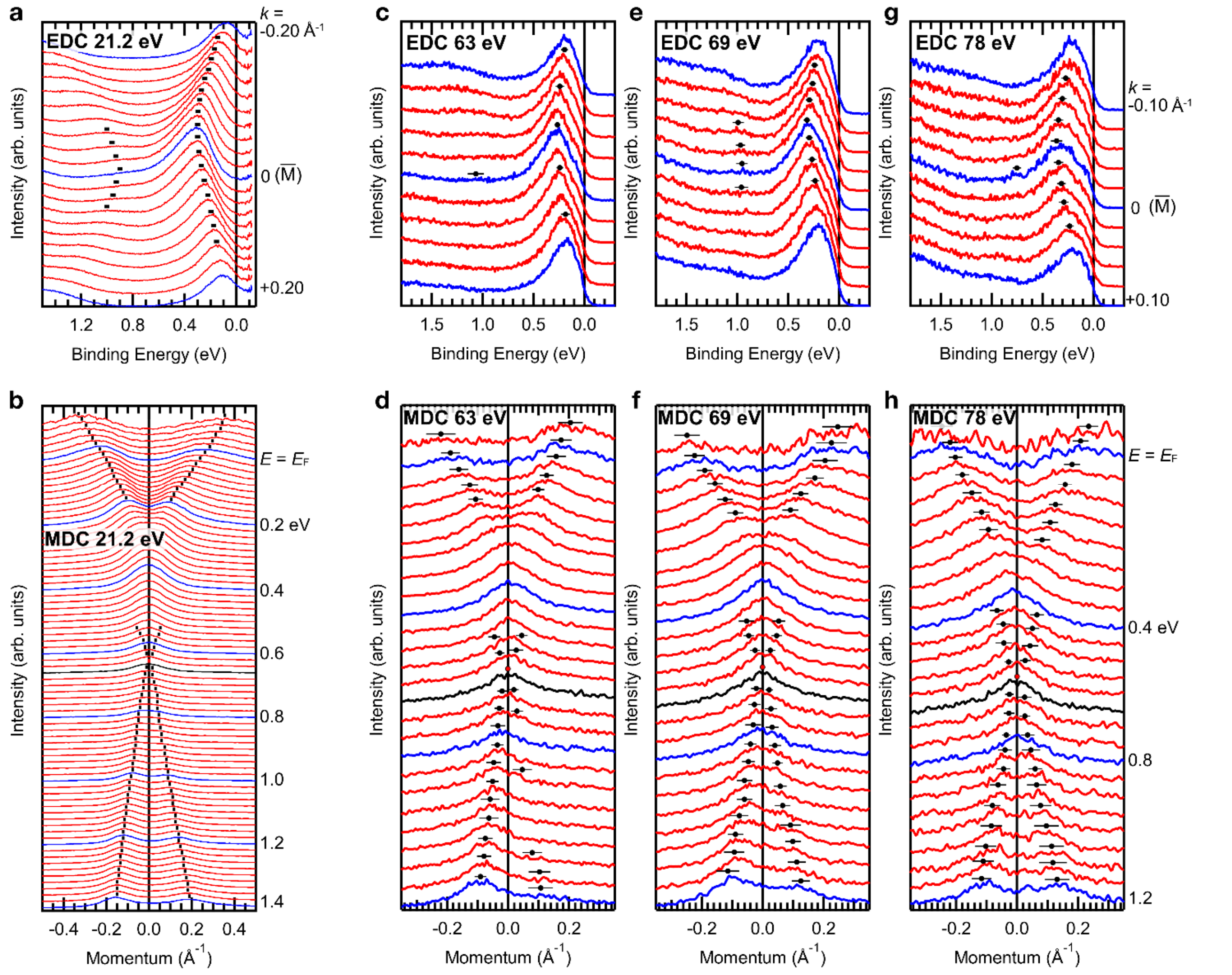

Supplementary Figure 7 | EDC and MDC of *hν*-dependent ARPES for 1*T*-$V_{0.90}Ti_{0.10}Te_2$. a, b, EDCs (**a**) and MDCs (**b**) taken with a He discharge lamp ($h\nu$ = 21.2 eV, 350 K). **c-h**, Same as **a** and **b**, but taken at 320 K with $h\nu$ = 63 (**c**, **d**), 69 (**e**, **f**), and 78 eV (**g**, **h**) circular polarization light. The markers in the figures denote the peak positions of EDCs/MDCs.

Supplementary Note 8: Setup of spin-resolved ARPES measurement.

Supplementary Figure 8a shows the geometry of spin-resolved ARPES measurements for multi-domain VTe_2 (see also Methods in the main text). To access the $\bar{K}_1 - \bar{M}_1 - \bar{K}_1/\bar{K}_1 - \bar{M}_2 - \bar{K}_2$ lines, the attached samples are rotated both around the polar (θ) and tilt (φ) axes. Two VLEED spin detectors can be selectively magnetized along (x, z) and (y, z) axes, respectively. The spin detector base (xyz) is converted to the sample base (XYZ) with the rotation matrices:

$$\begin{pmatrix} X \\ Y \\ Z \end{pmatrix} = \begin{pmatrix} 1 & 0 & 0 \\ 0 & \cos\varphi & -\sin\varphi \\ 0 & \sin\varphi & \cos\varphi \end{pmatrix} \begin{pmatrix} \cos\theta & 0 & \sin\theta \\ 0 & 1 & 0 \\ -\sin\theta & 0 & \cos\theta \end{pmatrix} \begin{pmatrix} x \\ y \\ z \end{pmatrix}. \quad (1)$$

The spin polarizations along x, y, z (*i.e.* P_x, P_y, P_z) are obtained by

$$P_{x,y,z} = \frac{1}{S_{\mathrm{eff}}} \frac{I^{+}_{x,y,z} - I^{-}_{x,y,z}}{I^{+}_{x,y,z} + I^{-}_{x,y,z}}, \quad (2)$$

where $I^{+}_{x,y,z}$ ($I^{-}_{x,y,z}$) is the raw spin-resolved ARPES spectra obtained with the target magnetized in the plus (minus) x, y, z directions. The spin-resolved spectra for spin-up (-down) components along x, y, z directions, $I^{\uparrow}_{x,y,z}$ ($I^{\downarrow}_{x,y,z}$), are obtained by

$$I^{\uparrow(\downarrow)}_{x,y,z} = \left(1 \pm P_{x,y,z}\right) \frac{I^{+}_{x,y,z} + I^{-}_{x,y,z}}{2} = \left(1 \pm P_{x,y,z}\right) \frac{I_{\mathrm{total}}}{2}, \quad (3)$$

where $I_{\mathrm{total}} = I^{+} + I^{-} = I^{\uparrow} + I^{\downarrow}$. The spin polarization and spin-resolved spectra in the XYZ sample base, $P_{X,Y,Z}$ and $I^{\uparrow(\downarrow)}_{X,Y,Z}$, can be obtained by using the following relations,

$$\begin{pmatrix} P_X \\ P_Y \\ P_Z \end{pmatrix} = \begin{pmatrix} 1 & 0 & 0 \\ 0 & \cos\varphi & -\sin\varphi \\ 0 & \sin\varphi & \cos\varphi \end{pmatrix} \begin{pmatrix} \cos\theta & 0 & \sin\theta \\ 0 & 1 & 0 \\ -\sin\theta & 0 & \cos\theta \end{pmatrix} \begin{pmatrix} P_x \\ P_y \\ P_z \end{pmatrix}, \quad (4)$$

$$\begin{pmatrix} I^{\uparrow(\downarrow)}_X \\ I^{\uparrow(\downarrow)}_Y \\ I^{\uparrow(\downarrow)}_Z \end{pmatrix} = \frac{I_{\mathrm{total}}}{2} \begin{pmatrix} 1 \\ 1 \\ 1 \end{pmatrix} \underset{(-)}{+} \frac{1}{2} \begin{pmatrix} 1 & 0 & 0 \\ 0 & \cos\varphi & -\sin\varphi \\ 0 & \sin\varphi & \cos\varphi \end{pmatrix} \begin{pmatrix} \cos\theta & 0 & \sin\theta \\ 0 & 1 & 0 \\ -\sin\theta & 0 & \cos\theta \end{pmatrix} \begin{pmatrix} I^{\uparrow}_x - I^{\downarrow}_x \\ I^{\uparrow}_y - I^{\downarrow}_y \\ I^{\uparrow}_z - I^{\downarrow}_z \end{pmatrix}. \quad (5)$$

The spin-resolved ARPES data in the main text (Figs. 5g-i) are collected with *s* polarized 21 eV light at $\varphi = 28°$ and $-4° \leq \theta \leq +4°$ setting. The sample orientation is arranged so that the sample axes X and Y become parallel to the $\mathbf{a}_m$ and $\mathbf{b}_m$ crystal axes for one of the 3 in-plane multi-domain. Supplementary Figures 8b and c show the raw $I_y^{+(-)}$ spectra and their subtraction ($I_y^{+} - I_y^{-}$) respectively at $\theta = \pm 4°$ (Note that after conversion $I_y^{\uparrow(\downarrow)}$ is displayed in Fig. 5i in the main text). We can find the slight plus/minus contrasts near the Fermi level in these raw data.

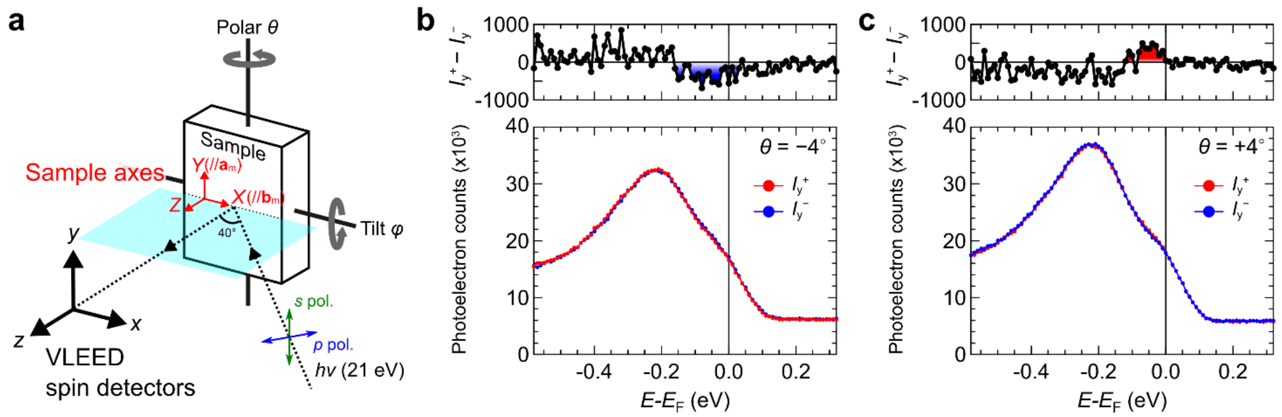

Supplementary Figure S8 | Experimental setup of spin-resolved ARPES. a, Schematic spin-resolved ARPES geometry. The attached samples are rotated around the polar (θ) and tilt (φ) axes to measure the band dispersion along $\overline{\mathrm{K}}_1-\overline{\mathrm{M}}_1-\overline{\mathrm{K}}_1/\overline{\mathrm{K}}_1-\overline{\mathrm{M}}_2-\overline{\mathrm{K}}_2$. **b,** The raw $I_y^{+(-)}$ spectra and their subtraction ($I_y^{+}-I_y^{-}$) at $\theta=-4^\circ$. **c,** Same as **b**, but at $\theta=+4^\circ$.

Supplementary Note 9:

ARPES images and EDCs/MDCs along $\overline{\mathrm{K}}_1-\overline{\mathrm{M}}_1-\overline{\mathrm{K}}_1/\overline{\mathrm{K}}_1-\overline{\mathrm{M}}_2-\overline{\mathrm{K}}_2$ for 1*T*''-VTe_2.

The details of ARPES images and their EDCs/MDCs along $\overline{\mathrm{K}}_1-\overline{\mathrm{M}}_1-\overline{\mathrm{K}}_1/\overline{\mathrm{K}}_1-\overline{\mathrm{M}}_2-\overline{\mathrm{K}}_2$ (15 K, multi-domain) for 1*T*''-VTe_2 are shown in Supplementary Fig. 9. Supplementary Figures 9a, e, i are the data recorded with $h\nu=54$, 61.5, 69 eV, respectively, which are similarly presented as Figs. 5j, k, l in the main text. Supplementary Figures. 9a, b, and c respectively show the ARPES image, EDCs, and MDCs taken with $h\nu=54$ eV (*s* polarized). For a clearer presentation, the MDCs near E_F are again displayed with different offsets in Supplementary Fig. 9d. The markers in Supplementary Fig. 9a are obtained by the peaks of EDCs and MDCs shown in Supplementary Figs. 9b-d. Supplementary Figures 9e-h and 9i-k similarly shows the sets of results recorded with $h\nu=61.5$ eV and 69 eV, respectively.

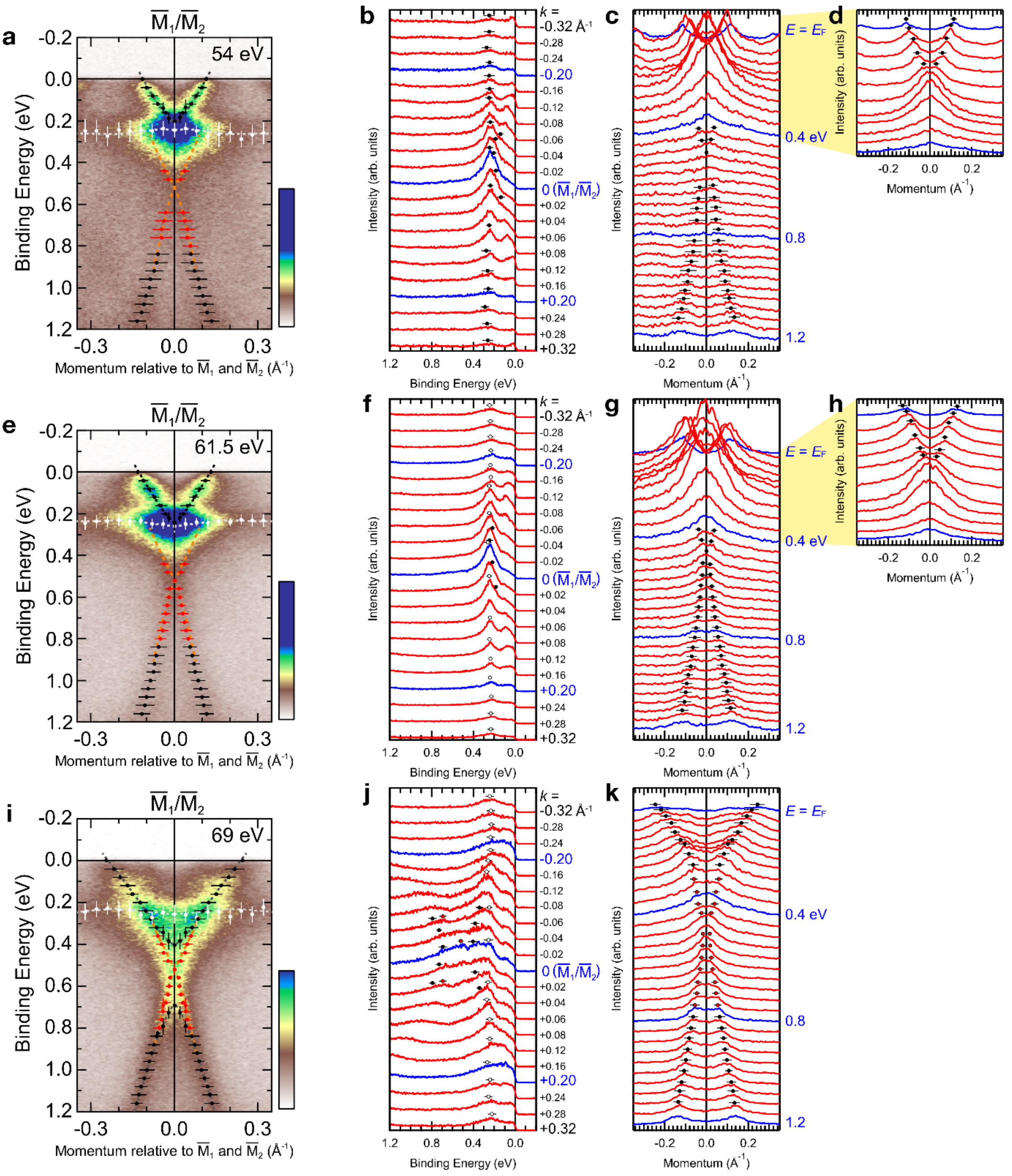

Supplementary Figure 9 | EDC and MDC of *hν*-dependent ARPES for 1*T*''-VTe_2. a, ARPES image recorded with with *hν* = 54 eV plotted with EDC/MDC peak positions. **b,** EDCs of **a** (integral width: 0.02 Å^{-1}). **c,** MDCs of **a** (integral width: 0.04 eV). **d,** Zoom-in of several MDCs of **a** around the Fermi level. **e-h,** Same as **a-d**, but taken with *hν* = 61.5 eV. **i-k,** Same as **a-c**, but taken with *hν* = 69 eV. The markers in the figures denote the peak positions of EDCs/MDCs.

Supplementary Note 10: Band calculation with V3*d* orbital weights.

Supplementary Figure 10 shows the V3*d*-orbital contributions in the electronic band structures of normal (1*T*) and CDW (1*T*") VTe_2 obtained by first-principles calculations. The definition of *XYZ* coordination for *d* orbitals is shown in Fig. 6a in the main text. Supplementary Figure 10a represents the BZ in the 1*T* phase; the blue arrows indicate the half-length reciprocal lattice vectors ($\mathbf{a}^*/2$, $\mathbf{b}^*/2$, $\mathbf{c}^*/2$). Supplementary Figure 10b displays the electronic band structures along the **k** path described as the red lines in Supplementary Fig. 10a. The color and size of circles respectively represent the *d* orbital component and its weight (Note that the small size of circle markers indicate the dominance of Te5*p*). Focusing on the M-K and L-H line, the band crossing the Fermi level is mainly derived from d_{XY} orbital (green circles). Therefore, by considering the three-fold rotation, each side of the triangular hole Fermi surface surrounding the K point (at $k_z = 0$) consist of one of three V3*d* orbitals, d_{YZ}, d_{ZX} and d_{XY} (see the cartoon of Fermi surfaces in Supplementary Fig. 10a). Supplementary Figure 10c shows the total DOS and PDOS for V3*d* and Te5*p*, indicating the dominant contribution of V3*d* in the vicinity of E_F. We note that this calculation of the pristine 1*T*-VTe_2 predicts stronger V3*d*-Te5*p* hybridization similar to the Ti-doped system $V_{0.90}Ti_{0.10}Te_2$ (Supplementary Note 3), thus the topological Dirac surface state is likely to be realized in 1*T*-VTe_2 as discussed in the main text.

Supplementary Figures 10d-f show the results for the CDW 1*T*" phase. For directly comparison with the result for the 1*T* case, the electronic band structures are plotted along the **k** path corresponding to those for the 1*T* results (see the red lines in Supplementary Fig. 10d). Looking at the M_1-K_1 line, the band dispersion with the dominant d_{XY}-orbital contribution cross E_F, which is similar to the 1*T* case. On the other hand, the flat dispersions with binding energy $E_B \sim 0.4$ eV on the M_2-K_2 line consist of d_{YZ} and d_{ZX} orbitals; see also the PDOS in Supplementary Fig. 10g. As discussed in the main text, we consider that the flat band of d_{YZ}/d_{ZX} orbitals originates in the vanadium trimers bonding.

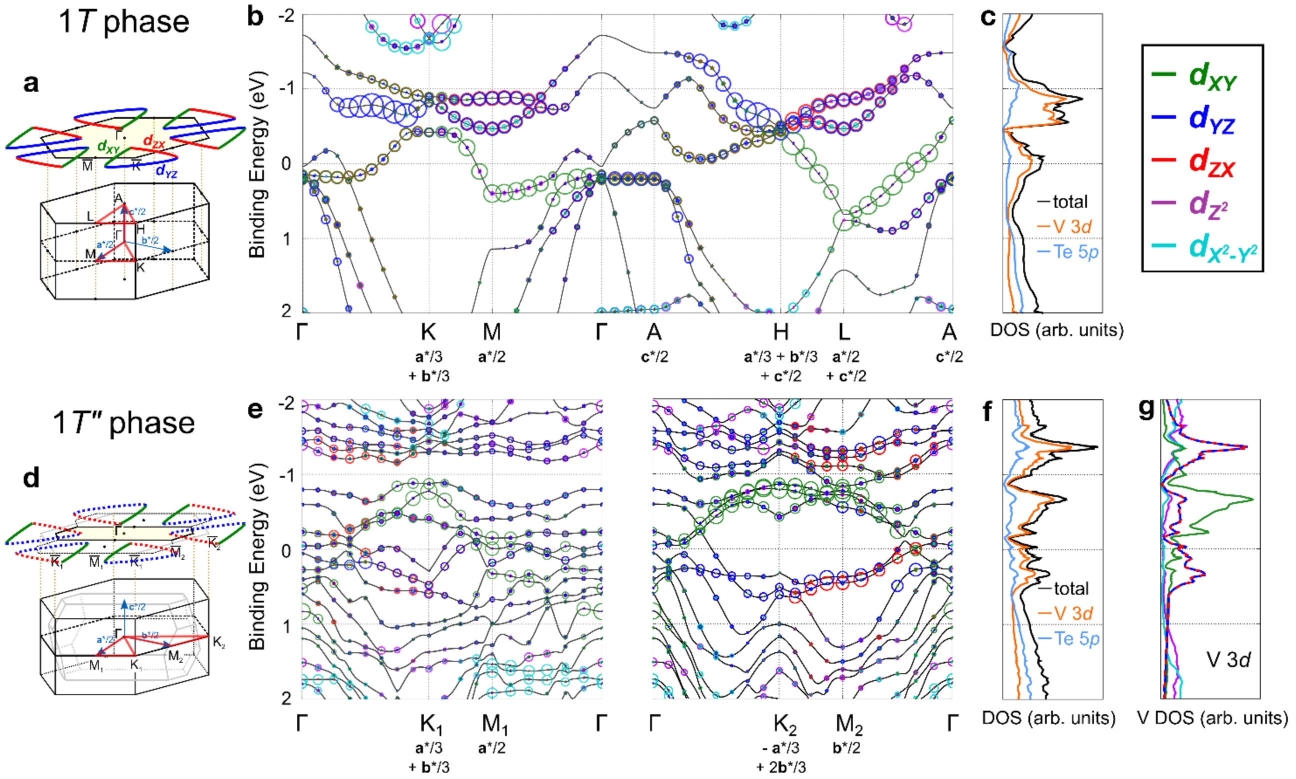

Supplementary Figure 10 | Electronic band structures of VTe_2 with V3*d* orbital contributions. a, BZ in the normal 1*T* phase. Cartoons of Fermi surfaces at $k_z = 0$ are also depicted. **b,** Electronic band structures of the 1*T* phase along the **k** path indicated by the red lines in **a**. The size and color of the circles indicate the relative weight of each *d* orbital. **c,** Total DOS and PDOS for V3*d* and Te5*p*. **d-f,** Same as **a-c**, but for the CDW 1*T*" phase. **g,** PDOS for each V3*d* orbital in the 1*T*" phase.

Supplementary Note 11: CDW effects on Te5*p* orbitals.

Supplementary Figures 11a and b show the calculated PDOS of 1*T*''-VTe_2, respectively for V3*d* (d_{XY}, d_{YZ}, d_{ZX}) and Te5*p* (p_X, p_Y, p_Z). They indicate that the flat band at the binding energy of 0.3 ~ 0.4 eV is formed by the hybridization of d_{YZ}/d_{ZX} and p_Z orbitals. Here we note that d_{YZ} and d_{ZX} well hybridize with p_Z in the edge-sharing VTe_6 octahedral network, whereas the overlap for d_{XY} and p_Z is negligibly small (see Supplementary Fig. 11c).

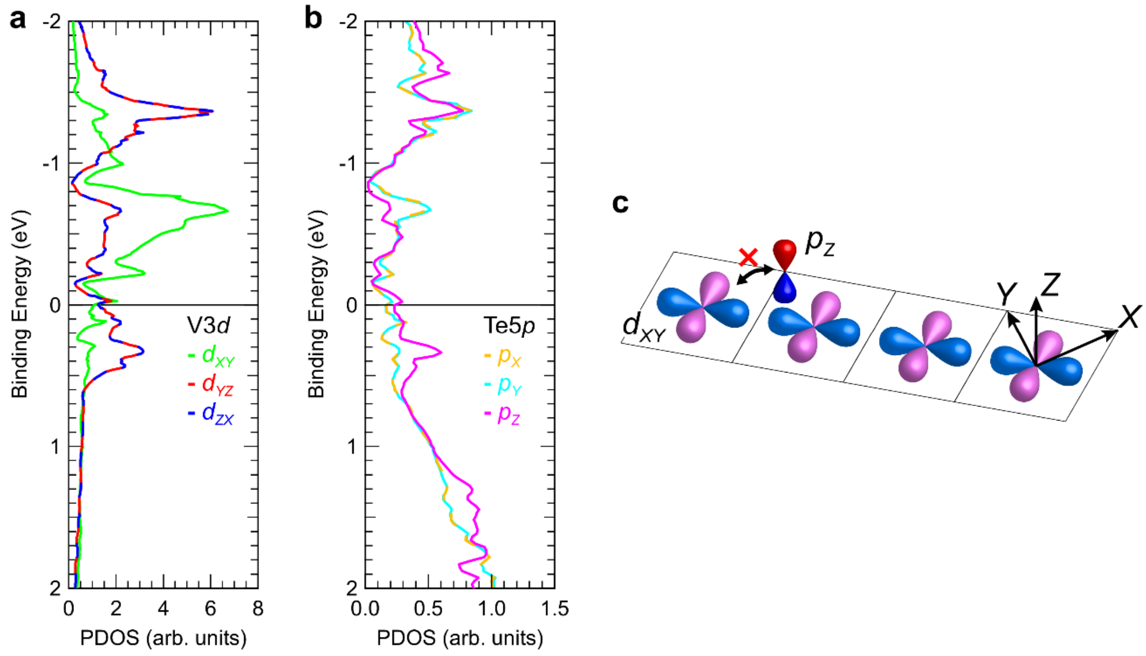

Supplementary Figure 11 | PDOS of 1*T*''-VTe_2. a, PDOS for V3*d* (d_{XY}, d_{YZ}, d_{ZX}). **b,** PDOS for Te5*p* (p_X, p_Y, p_Z). **c,** Schematic drawing of p_Z orbital along the d_{XY} σ-bonding.

Supplementary Note 12: Band unfolding in 1*T*''-VTe_2.

We have calculated the electronic structures of VTe_2 with the 1*T*''-CDW formation by using the so-called unfolding method, which enables us to directly compare the ARPES results with first principles calculations [4, 5]. Supplementary Figures 12b and c show the results along the K_1-M_1-K_1 and K_1-M_2-K_2 line (see the red and blue arrows in Supplementary Fig. 12a), which qualitatively reproduce the ARPES results for a single domain region (Figs. 5b and 5d in the main text). We note that the surface state observed in ARPES measurement (Fig. 5b) does not appear in this bulk calculation. Supplementary Figure 12d displays the constant energy contours obtained by the first-principles calculations (upper panels) and single-domain ARPES measurement (lower panels) at several binding energies (E_B). The both results exhibit the quasi-one-dimensional electronic structure.

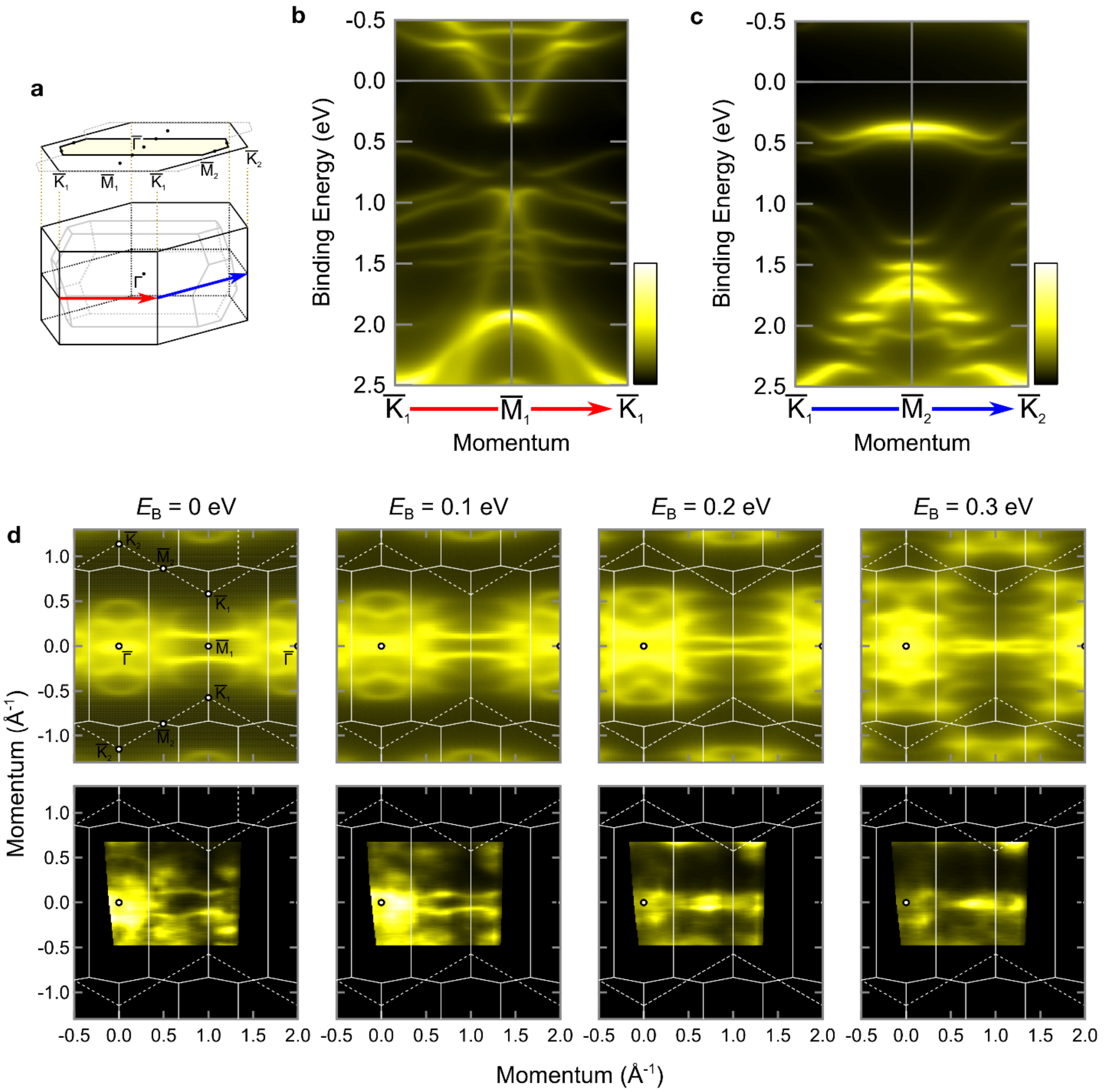

Supplementary Figure 12 | Calculated electronic structures of 1*T*"-VTe_2 by the unfolding method. **a,** BZ of the normal 1*T* phase (black lines) and the CDW 1*T*" phase (gray lines). **b, c,** The calculated band structures along the ***k*** paths depicted in **a** by red and blue arrows, respectively. **d,** Constant energy contours obtained by the calculations (upper panels) and experiments (single-domain, lower panels) at E_B = 0 (Fermi level), 0.1, 0.2, and 0.3 eV.

Supplementary References

[1] Bronsema, K. D., Bus, G. W. & Wiegers, G. A. The crystal structure of vanadium ditelluride, $V_{1+x}Te_2$. *J. Solid State Chem.* **53,** 415–421 (1984).

[2] Battaglia, C. *et al*. Fermi-surface-induced lattice distortion in $NbTe_2$. *Phys. Rev. B* **72**, 195114 (2005).

[3] Chen, C. *et al*. Trimer bonding states on the suface of the transition-metal dichalcogenide. *Phys. rev. B* **98**, 195423 (2018).

[4] Ku, W., Berlijn, T., & Lee, C.-C. Unfolding First-Principles Band Structures, *Phys. Rev. Lett.* **104**, 216401 (2010).

[5] Lee, C.-C., Yamada-Takamura, Y., & Ozaki, T. Unfolding method for first-principles LCAO electronic structure calculations, *J. Phys.: Cond. Matter* **25**, 345501 (2013).
